# Long-term potentiation and long-term depression are both impaired after *in vitro* stretch injury measured with stretchable microelectrode arrays

**DOI:** 10.1088/2057-1976/adea7e

**Published:** 2025-07-14

**Authors:** Mary Kate R Dwyer, Isabella Polsfuss, Keondre Herbert, Nevin Varghese, Barclay Morrison

**Affiliations:** Department of Biomedical Engineering, Columbia University, New York, NY, 10027, United States of America

**Keywords:** traumatic brain injury, long-term potentiation, long-term depression, serial recordings, stretchable microelectrode arrays, plasticity

## Abstract

*Objective*. Traumatic brain injury (TBI) is a prevalent injury that can lead to long term deficits in memory and cognition. Predicting which patients will have long lasting memory issues following mild TBI is challenging. *Approach.* Organotypic hippocampal slice cultures were biaxially stretched to model a TBI. In this *in vitro* model, stretchable microelectrode arrays were embedded within the culture substrate to both deform the adhered culture and record neural signals, which are indicators of neuronal health and network connectivity. Multiple spontaneous and evoked recordings were obtained while maintaining sterility to study and modulate the electrophysiological response to injury. *Main results*. In the first set of experiments, neural signals were measured 2 and 24 h after stretch injury. Bursting activity increased 2 h after injury but returned to baseline by 24 h. However, 24 h after injury, both long-term potentiation (LTP) and long-term depression (LTD) were impaired. In another experiment, LTP was induced multiple times, both 24 h before and 24 h after injury, to study how the state of the pre-injury network affected electrophysiological outcome after injury. We provide preliminary evidence that induction of LTP before injury to increase synaptic strength was detrimental to neuronal plasticity (LTP) after injury. Future studies can use the stretchable microelectrode arrays and our induction paradigm to test if induction of LTD, a weakening of synaptic strength, could increase resiliency to injury. *Significance.* This research begins to examine the role of pre-injury network connectivity and synaptic strength on post-traumatic electrophysiological outcomes, which may increase understanding of the determinants of heterogeneous clinical outcomes in mild TBI.

## Introduction

Traumatic brain injury (TBI) is a major health concern worldwide. It is estimated that over 1.3 million people in the U.S. are treated for TBI and released without hospitalization annually, likely indicating a mild injury [1]. Injuries requiring hospitalization are generally more severe, but even mild TBI (mTBI) can cause lasting symptoms including memory loss and learning difficulties in some patients [2]. Neuroimaging techniques and multiple biomarkers are used clinically to grade injury severity [3, 4]. The relevance of these biomarkers to patient outcome is debated [3, 5]. Computed tomography (CT) imaging is considered the gold standard for injury diagnosis, but only 20% of mTBI patients have damage detectable by CT [6]. Additionally, the risk factors for patients that will experience lasting changes to brain function following mTBI are not well understood [7]. This gap in diagnosis prevents clinicians from reliably predicting the development of post-concussive syndrome. Patients with mTBI may have cognitive deficits within the first 24 h after injury, including deficits in working memory and amnesia, which may progress to chronic issues [8, 9]. Improved understanding of the acute factors that lead to poor outcome could lead to improved care following TBI.

Serial recordings over multiple days with microelectrode arrays (MEAs) have been feasible since the late 1990s [10–12]. However, traditional MEAs are made of glass and so, in the context of TBI, serial recordings both before and after injury are not possible. Stretch injury is a common model of TBI in which samples are adhered to a silicone substrate that is deformed to model TBI [13–15]. The organotypic hippocampal slice culture (OHSC) maintains cell types found in the parenchyma of the rat brain, including neurons, astrocytes, microglia, and oligodendrocytes in a three-dimensional matrix that maintains the structure of the hippocampus and has been used to study neuronal plasticity and spontaneous activity [16–19].

Stretchable microelectrode arrays (SMEAs) have been used previously to measure spontaneous activity over time from the same tissue before and after injury, where bicuculline administration elucidated differences in spontaneous network activity [20–22]. Recent work with SMEAs classified networks of dissociated cortical neurons by their strength before injury, testing the hypothesis that strongly connected networks might respond differently to injury than weakly connected networks. However, no changes in the chosen metrics were found after injury [23]. Although still an open question, classifying networks or even modifying networks before injury may lead to a better understanding of the variable prognoses following TBI. The SMEAs combined with the OHSCs provide an exciting experimental approach to manipulate electrophysiological activity of the well-studied hippocampal tri-synaptic circuit before injury and to study the effect on functional outcome after injury.

Long-term potentiation (LTP), a cellular correlate of learning and memory in which synaptic connections are strengthened after high frequency electrical stimulation (HFS), is disrupted after TBI [24–28]. In contrast, long-term depression (LTD), a weakening of synaptic connections following low frequency stimulation (LFS), has been studied less, with conflicting reports from *in vivo* studies whether LTD is disrupted after TBI [25, 29–32]. Aspects of the secondary injury cascade, such as excitotoxicity, are thought to impair both LTP and LTD, two forms of Hebbian plasticity [33, 34]. Several studies have also identified changes in spontaneous activity due to TBI [35–37], and induction of plasticity may affect spontaneous activity [36]. The OHSC model enables the study of the direct effect of mechanical deformation on plasticity and spontaneous activity, without systemic influences, and may provide additional insights into LTD after mechanical injury.

It is hypothesized that the current state of a neural network affects how it will respond to stimuli and that specific cellular mechanisms drive stability following external stimuli [38–41]. In support of this hypothesis, transcranial direct current stimulation leads to better outcomes when there is a high degree of white matter connectivity [42]. TBI is a stimulus that alters network connectivity [22, 33, 43, 44]. Neurons have the ability to regulate their electrical activity through homeostatic plasticity, such that cells may respond to increased excitation following TBI by decreasing their excitability. Decreased excitability can be achieved by decreasing ion conductance or the number of synapses [45]. Because homeostatic plasticity may share mechanisms with Hebbian plasticity, strong homeostatic plasticity has been suggested decrease the ability to induce Hebbian plasticity [46].

Changes in network connectivity following TBI can be partially attributed to excitotoxicity, which is an overexposure of neurons to glutamate, that can change neuron excitability [47]. The distribution of glutamate receptors before injury affects network plasticity following injury, with glutamate receptors contributing to uncontrolled activity after injury [48]. Additionally, in an *in vitro* model of cortical neurons, networks with lower initial synchronization were more resilient to mechanical injury [49]. Therefore, it is possible that network strength and activity before injury may affect how the network responds to TBI. In fact, age-associated changes in network strength may be why age is a major, negative, determinant of outcome after TBI [50].

Induction of LTP or LTD alters the current network state to be either more or less synchronous and responsive to a stimulus. We hypothesize that the network state at the time of injury will affect the outcome and aim to provide evidence as to whether a synchronous state induced pre-injury by inducing LTP leads to altered recovery after trauma. Schroeder *et al* found that inhibiting the metabotropic glutamate receptor 5 (mGluR5), which can affect synaptic strength, before injury prevented changes to connectivity after injury [44]. If the neuroprotective mechanism of mGluR5 inhibition is through changes to network strength and glutamate signaling, then other strategies to alter the network connectivity may also be neuroprotective.

Here we assess alterations in spontaneous and evoked responses following injury in an *in vitro* model. We present methods and preliminary data using serial recordings to study TBI. Using these methods, OHSCs were stimulated before injury to alter the neuronal network to ask whether the pre-injury state altered the time course of recovery post-injury. This experimental paradigm could shed light on the functional mechanisms that contribute to the speed of recovery after TBI.

## Methods

### Organotypic hippocampal slice culture

All animal procedures were approved by the Columbia University Institutional Animal Care and Use Committee (IACUC) and in accordance with AAALAC-International standards. Culture methods have been described in detail previously [27, 28, 51, 52]. To prepare the silicone culture substrate and improve adhesion of the OHSCs, the SMEA was coated with a mixture of poly-L-lysine (0.32 mg ml^−1^; Millipore Sigma) and laminin (0.067 mg ml^−1^; Gibco). 24 h later, the poly-L-lysine and laminin solution was replaced with Neurobasal medium supplemented with 2 mM GlutaMAX (ThermoFisher), 1X B27 (ThermoFisher), 10 mM HEPES (Millipore Sigma), and 25 mM D-glucose (ThermoFisher). 18–24 h after Neurobasal medium was added, the medium was removed. Hippocampi of P8—10 Sprague-Dawley rats were isolated and sliced to a thickness of 400 μm with a McIlwain tissue chopper (Ted Pella). Under a dissecting microscope, a hippocampal slice was placed onto the electrode array in the center of each SMEA, and Neurobasal medium was added. SMEAs were then rocked in an incubator maintained at 37 °C and 5% CO_2_. Every 2–3 days, half of the medium was replaced with fresh serum containing medium (50% minimum essential medium (Millipore Sigma), 25% Hank’s balanced salt solution (Millipore Sigma), 25% heat inactivated horse serum (ThermoFisher), 2 mM GlutaMAX, 25 mM D-glucose, and 10 mM HEPES). Cultures were kept for at least 10 days *in vitro* before use in experiments.

### Cell death measurement

Cell death was quantified before experimentation with propidium iodide (PI; Life Technologies) to verify culture health. Prior to imaging, cultures were incubated in 2.5 μM PI in serum-free medium (75% minimum essential medium, 25% Hank’s balanced salt solution, 2 mM GlutaMAX, 25 mM D-glucose, and 10 mM HEPES) for 30 min. Images were taken with an Olympus IX81 microscope with excitation of 568/24 nm (peak/width) and emission at 610/40 nm with a Hamamatsu C11440 digital camera. Cell death was measured with Metamorph (Molecular Devices) as the percent area of the hippocampus above a predetermined fluorescent threshold. Consistent with previous studies, OHSCs with >5% cell death prior to injury were excluded from the study [53].

### Injury

Healthy OHSCs were injured similarly to previously described methods [28, 51, 52]. Briefly, medium was removed from a well, which was clamped onto the injury device (BMSEED; bmseed.com). Under feedback control, the SMEA was rapidly lowered onto a hollow circular indentor with a linear actuator, stretching the silicone substrate and the adhered OHSC. The silicone substrate of the SMEA has a Young’s Modulus of 2 MPa. This technology has been designed with the goal of maintaining low impedance after thousands of repetitive stretches, by fabricating the electrodes from microcracked golds [20]. Each well was immediately returned to the incubator with serum containing medium. Injury biomechanics (tissue strain and strain rate) were verified with digital image correlation from high-speed videos taken at 1000 frames per second (Phantom or BMSEED) of the injury event using a custom MATLAB (Mathworks) script. The average strain was 14.68 ± 1.34% (mean ± SEM) with an average strain rate of 14.51 ± 1.63 s^−1^.

### Electrophysiological recordings

All electrophysiology data was collected before (pre) or after (post) stretch injury. No electrophysiology data was collected during stretch injury, only when the SMEA was in a relaxed state. All electrophysiological activity was recorded with a 28-channel SMEA (BMSEED) through the MEASSuRE-X system (BMSEED) sampling at 20 kHz with a 7.6 kHz antialiasing filter. The electrodes cover a recording area of 2.32 mm by 1.72 mm and sit in a circular well with a diameter of 25.4 mm. The electrodes themselves are gold coated with lead-free platinum black. They measure 100 μm by 100 μm with 400 μm center-to-center spacing between electrodes in the same row and 447 μm between electrodes in adjacent rows. All data were then digitally filtered through a 0.2–1000 Hz bandpass Butterworth filter in MATLAB (Mathworks).

Electrode impedance (Z) measurements were collected through the MEASSuRE-X system using a 1000 Hz sine wave at 3 current levels (38 nA, 3.8 nA, and 0.38 nA). In terminal recordings, OHSCs were perfused with artificial cerebral spinal fluid (aCSF) containing 125 mM NaCl, 3.5 mM KCl, 26 mM NaHCO_3_ (ThermoFisher), 1.2 mM KH_2_PO_4_, 2.4 mM CaCl_2_, 1.3 mM MgCl_2_, 10 mM HEPES, and 10 mM D-glucose, which was oxygenated with 95% O_2_/5% CO_2_ and warmed to 37 °C. All other reagents were purchased from Millipore Sigma.

In serial recordings where sterility was maintained, OHSCs were submerged in oxygenated and warmed serum containing medium; electrophysiological activity was recorded once before injury and once after. A 3D-printed cap (surgical guide resin, FormLabs) with a 12.7 μm thick fluorinated ethylene-propylene membrane (ALA Scientific) was placed over the SMEA to allow gas exchange while preventing contamination.

### Spontaneous electrophysiological recordings

Spontaneous local field potentials (LFPs) were recorded through the SMEAs for 5 min in serum containing medium. Recordings were saved and filtered in MATLAB with a recursive least squares filter using the Ag/AgCl ground [53]. Electrodes were only used if they had an impedance of <1 MOhm before and after stretch injury. LFPs were identified using a multi-resolution Teager energy operator (m-TEO) [54], as described previously [22], using k-values of [15, 30, 45]. Electrophysiological events were identified as being above the 97.5th percentile of the baseline m-TEO (approximately 2 standard deviations above the mean of the baseline), and any events shorter than 1.5 ms were excluded. Firing rate was calculated as the total number of events divided by the length of the recording, and average magnitude of events was calculated using the peak-to-peak magnitude.

Additionally, the start time of each event was used to determine the correlation of activity between electrode pairs, as has been published previously [53]. The correlation between electrodes was calculated using the spike time tiling coefficient (STTC) (equation (1)) [55].

\begin{eqnarray*}{{STTC}}_{{ij}}=0.5* \left(\frac{{P}_{i}{\mathrm{-}}{T}_{j}}{1{\mathrm{-}}{P}_{i}{T}_{j\,}}+\frac{{P}_{j}{\mathrm{-}}{T}_{i}}{1{\mathrm{-}}{P}_{j}{T}_{i\,}}\right)\end{eqnarray*}

T_i_ and T_j_ were the sums of the total duration of events in electrodes *i* and *j* (respectively) divided by the total recording time. We used a correlation window of 1.5 ms. P_i_ was the number of LFPs found in electrode *i* that were within the correlation window of a LFP in electrode *j.* P_j_ was calculated similarly. Connectivity was calculated as the average of the STTC matrix of all electrodes that were in contact with the tissue.

Bursting analysis was performed using the start times of the events; within an electrode, a burst of activity was defined as 5 events within 100 ms [56]. Once bursts were identified, they were characterized by burst length (number of events) and the number of bursts that occurred per minute for each electrode. Percentage of events within bursts was calculated as the percentage of events that were included within an identified burst compared to all events identified.

### Stimulus response curves

Stimulus response (SR) curves of the Schaffer collateral (SC) pathway were generated using 2 bi-polar electrodes in constant current mode from the arrays with bi-phasic pulses with 0.1 ms per phase with the positive phase first in one electrode and the negative first in the other [26, 27, 53]. Stimulations increased from 0–80 μA in 10 μA increments (represented by S), and the response (represented by R) measured as the peak-to-peak evoked activity. The evoked response was fit to a sigmoidal function (equation (2)) [26].

\begin{eqnarray*}R\left(S\right)=\frac{{R}_{\max }}{1+{e}^{m({I}_{50}-S)}}\end{eqnarray*}

I_50_ was the current needed to induce half of the maximal response (R_max_), and m was proportional to the slope of the sigmoid [26].

### Terminal LTP recordings

In our study, experiments that ended after a single recording session were designated terminal because the OHSC was perfused with aCSF, thereby breaking sterility. The OHSC was stimulated once a minute for 30 min at I_50_ to record a baseline response. LTP was induced with 3 rounds of 100 pulses at 100 Hz (HFS), separated by 10 seconds, at the I_50_. The I_50_ was determined immediately after collection of the SR curve. The value of I_50_ varied between recordings but was always within the range of 5–30 μA. The OHSC was then stimulated once a minute for another hour at the I_50._ LTP was calculated as the percent change of the magnitude of the response in the *cornu ammonis* 1 (CA1) from the last 10 min of baseline to the response 50–60 min after LTP induction. Electrodes were included in the average if their baseline response was less than the estimated R_max_ for the electrode, and the average baseline response was above 100 μV. All data were analyzed in MATLAB (Mathworks) using custom scripts.

### Terminal LTD recordings

The OHSC was stimulated once a minute for 30 min at I_50_ to record a baseline response. LTD was induced with 900 pulses at 1 Hz (LFS) at the I_50_. The I_50_ was determined immediately after the collection of the SR curve. The value of I_50_ varied between recordings but was always within the range of 5–30 μA. The OHSC was then stimulated once a minute for another hour at the I_50._ LTD was calculated as the percent change of the magnitude of the response in the CA1 from the last 10 min of baseline to the response 50–60 min after LTD induction. Electrodes were included in the average if their baseline response was less than the estimated R_max_ for the electrode and the average baseline response was above 100 μV. All data were analyzed in MATLAB (Mathworks) using custom scripts.

### Serial LTP recordings

In the case where multiple recordings were measured over days, naïve OHSCs were used to generate an SR curve and stimulated at I_50_ to create a baseline_._ In the serial recordings, the baseline was abbreviated so that the OHSC was stimulated once every 30 seconds for 5 min [57–59]. Cultures then received either no stimulation or HFS stimulation to induce LTP. 1 h after the baseline recording and plasticity induction, samples were returned to the recording device to record the response at the previously determined I_50_. 24 h later, samples were either stretch-injured or received a sham-injury. 24 h after injury or sham-injury, an SR curve was generated again, and baseline and LTP recordings were taken at the I_50_. Electrodes in CA1 were included in the average if their baseline response was less than the estimated R_max_ for the electrode, the average baseline response was above 100 μV, and the impedance of the electrode was approximately equal before and after LTP, such that good contact was made during both parts of the experiment. All data were analyzed in MATLAB (Mathworks) using custom scripts.

### Statistical analysis

GraphPad Prism was used for all statistical analysis. Student’s t-tests were used to determine the significance of differences in terminal LTP and LTD experiments. A repeated measures two-way ANOVA was used to assess differences in spontaneous recordings, followed by uncorrected Fisher’s LSD post-hoc test to identify differences between groups. Unpaired one-tailed Student’s t-tests were used to determine differences between groups in the serial LTP data. For comparisons within a serial LTP group, a paired one-tailed Student’s t-test was used. Significance was set to p < 0.05 for all experiments.

## Results

### Spontaneous activity was altered 2 h after moderate injury

We confirmed that the electrodes remained functional after stretch injury and that there was no significant change in the impedance of the electrodes due to stretch. The resistance of electrodes in the SMEAs used for serial stretch was 0.28 ± 0.16 MOhm (mean ± SD) before stretch and 0.19 ± 0.09 MOhm after stretch, which was not statistically different (p=0.97, paired, one-tailed t-test).

**Figure 1.**
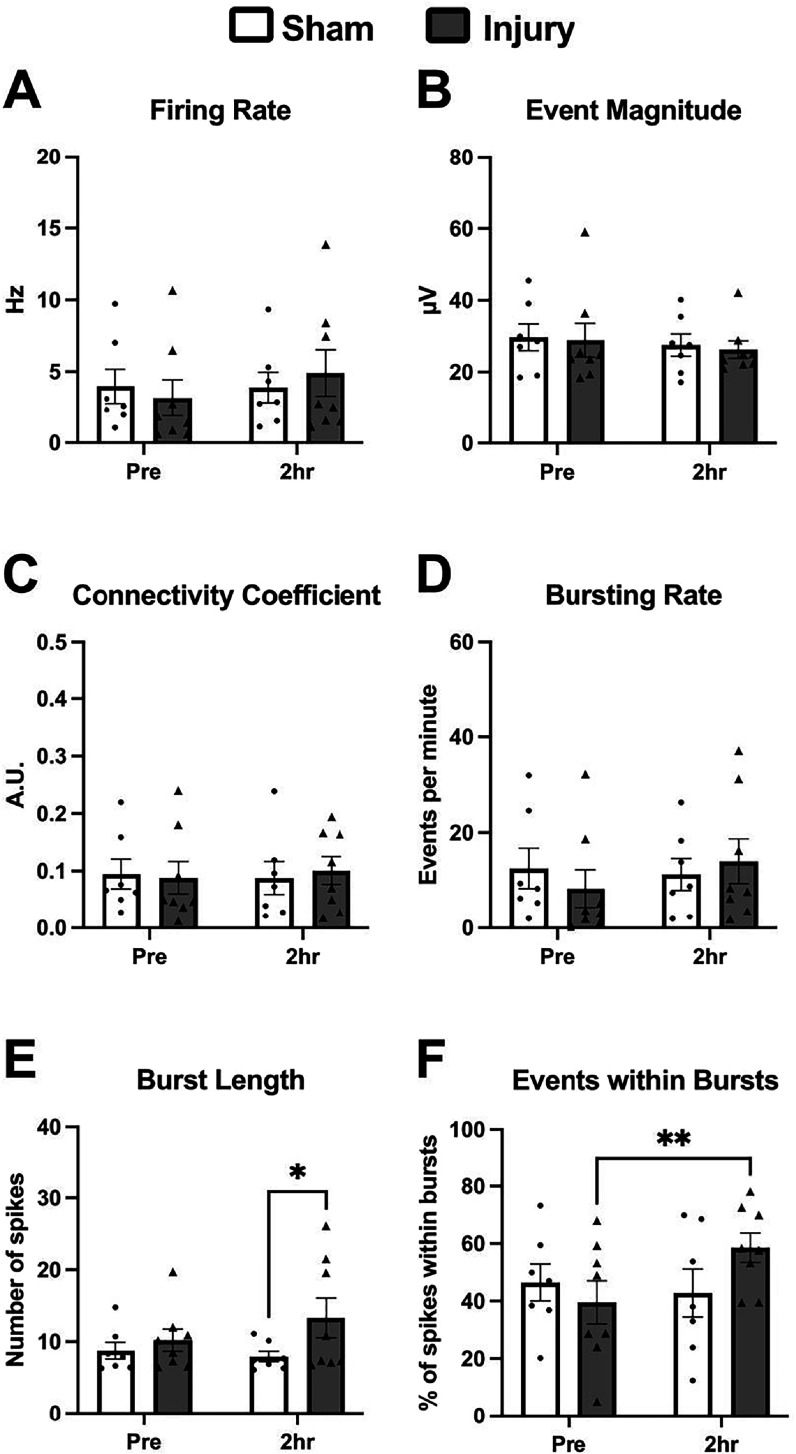
Spontaneous activity was altered 2 h after stretch injury. Injury did not affect firing rate (A), average event magnitude (B), connectivity coefficient (C) or bursting rate (D). Average burst length (E) was increased compared to the sham group following injury. The percentage of events found within bursts (F) increased compared to the pre-injury control. Mean ± SEM; N = 7–8. Repeated measures two-way ANOVA with uncorrected Fisher’s LSD test *p < 0.05, **p < 0.01.

Two hours after injury, there were no differences in firing rate, average event magnitude, or the connectivity coefficient. However, injury increased bursting behavior. 2 h after injury, the average burst length was increased compared to sham samples (p < 0.05, figure 1(E)). Additionally, the percentage of events within bursts increased in the injured group compared to the pre-injury recording (p < 0.01, figure 1(F)).

**Figure 2.**
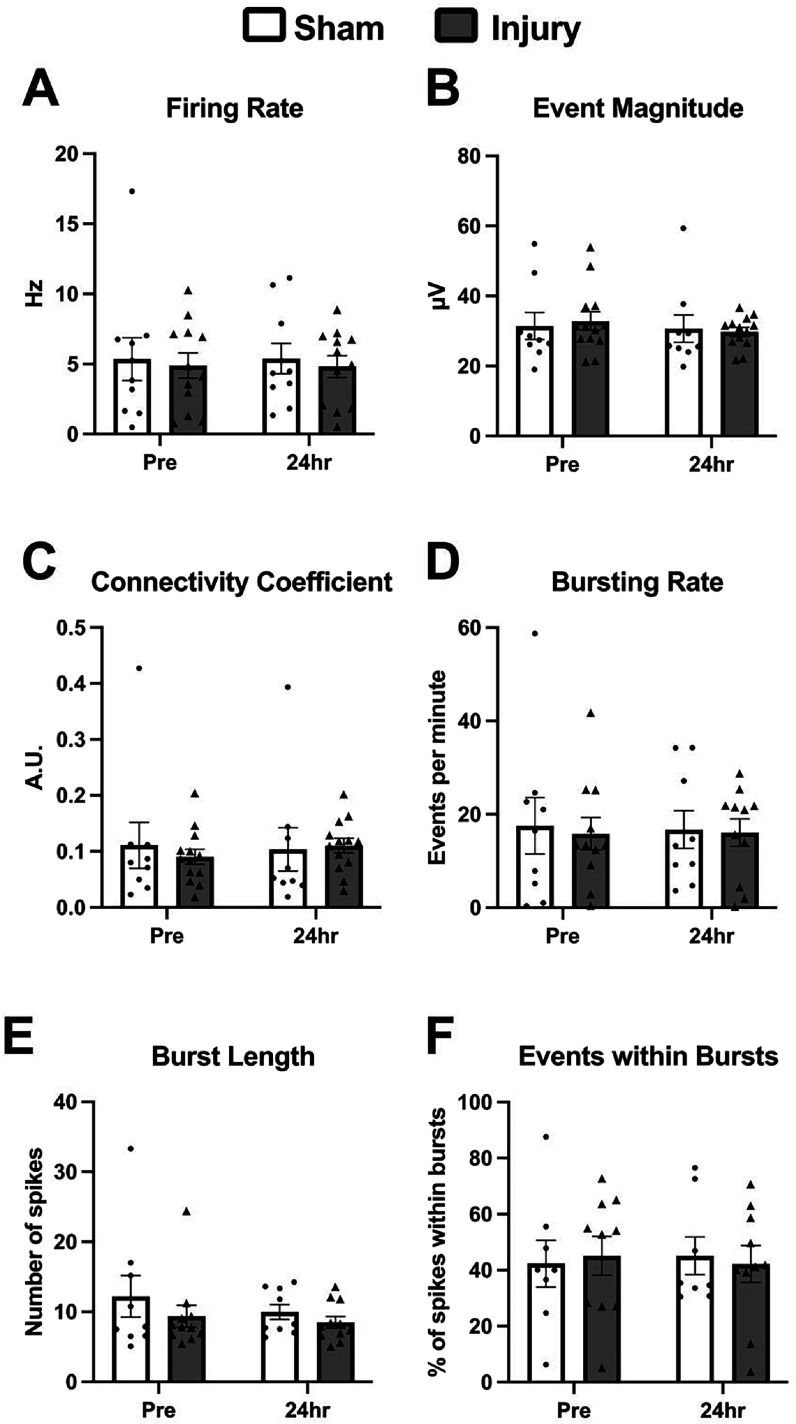
Spontaneous activity was not different 24 h after stretch injury, either compared to the pre-injury recording or between sham and injured samples. Injury did not affect firing rate (A), average event magnitude (B), the connectivity coefficient (C), bursting rate (D), average burst length (E), or the percentage of events found within bursts (F). Mean ± SEM; N = 10–12.

At 24 h after injury, there were no differences in the metrics of spontaneous activity assessed. Firing rate, average event magnitude, and the connectivity coefficient were not different between groups, and there were no differences compared to the values recorded before injury (figure 2). In addition, metrics assessing bursting behavior, including bursting rate, average burst length, and the percentage of events included within bursts, were not changed 24 h after injury.

### LTP decreased 24 h after moderate injury

**Figure 3.**
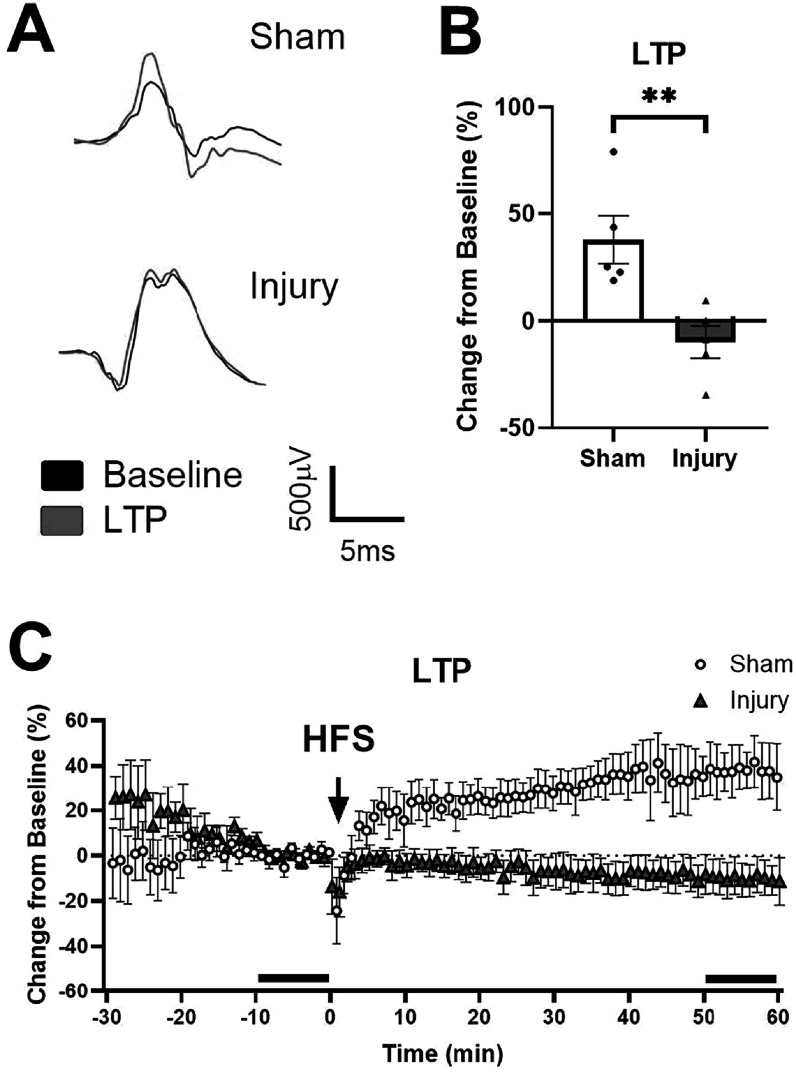
LTP-induction 24 h after stretch injury or sham-injury. (A) Representative traces from electrodes in CA1 of sham and injured samples before and after induction of LTP. (B) Stretch injury significantly reduced potentiation. Bar graph depicts the percent change of the average of the last 10 min of LTP normalized to the last 10 min before induction. (C) Longitudinal graph of the 30 min before induction with high frequency stimulation (HFS) and the 60 min after induction of LTP. The last 10 min of baseline and LTP are noted with a black bar above the X-axis. Mean ± SEM; N = 5; **p < 0.01 compared to sham using Student’s t-test.

24 h after injury, we confirmed that electrodes that had been stretched were functional and able to both record and stimulate the OHSCs. We tested the effects of a mild stretch injury on LTP-induction 24 h after injury. In the terminal LTP experiment, there were no changes in the R_max_ or I_50_ before induction (data not shown). LTP potentiation was significantly lower in the injured group than the sham (p < 0.01; figure B). In the sham group, the evoked response increased shortly after induction and stabilized by 1 h. In the injured group, the evoked response remained near pre-induction levels (figure 3(C)).

### LTD decreased 24 h after moderate injury

**Figure 4.**
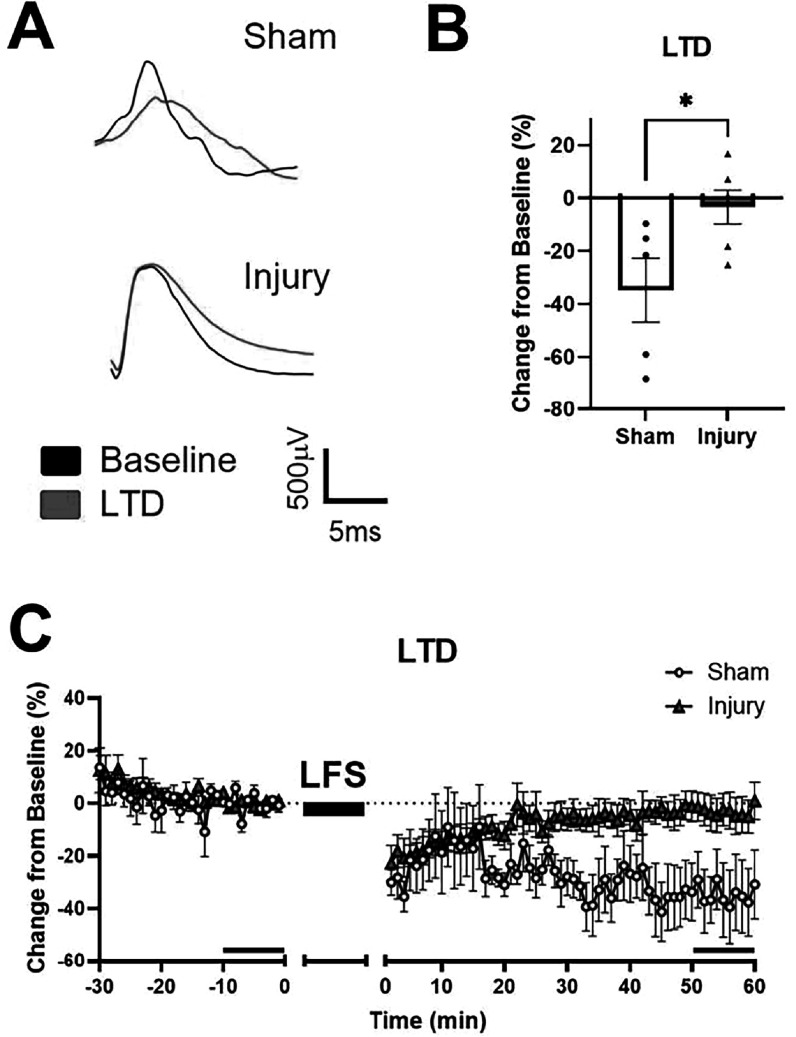
LTD-induction 24 h after stretch injury or sham-injury. (A) Representative traces from electrodes in CA1 of sham and injured samples before and after induction of Ltd (B) Stretch injury significantly reduced depression. Bar graph depicts the average of the last 10 min of LTD normalized to the last 10 min before induction. (C) Longitudinal graph of the 30 min before induction with low frequency stimulation (LFS), the 15 min of LFS, and the 60 min after induction of Ltd The last 10 min of baseline and LTD are noted with a black bar above the X-axis. Mean ± SEM; N = 5–6; *p < 0.05 compared to sham using Student’s t-test.

In addition to induction of LTP, we tested LTD-induction following mild stretch injury. As in the LTP experiment, there were no differences in R_max_ or I_50_ before induction of LTD (data not shown). In the terminal LTD experiment, there were significant deficits in LTD in the injured group at 24 h after stretch injury (p < 0.05, figure 4(B)). The response of the injured samples decreased briefly after induction but returned to near baseline. However, the response of the sham samples continued decreasing before stabilizing by 1 h (figure 4(C)).

### LTP was inducible without perfusion

**Figure 5.**
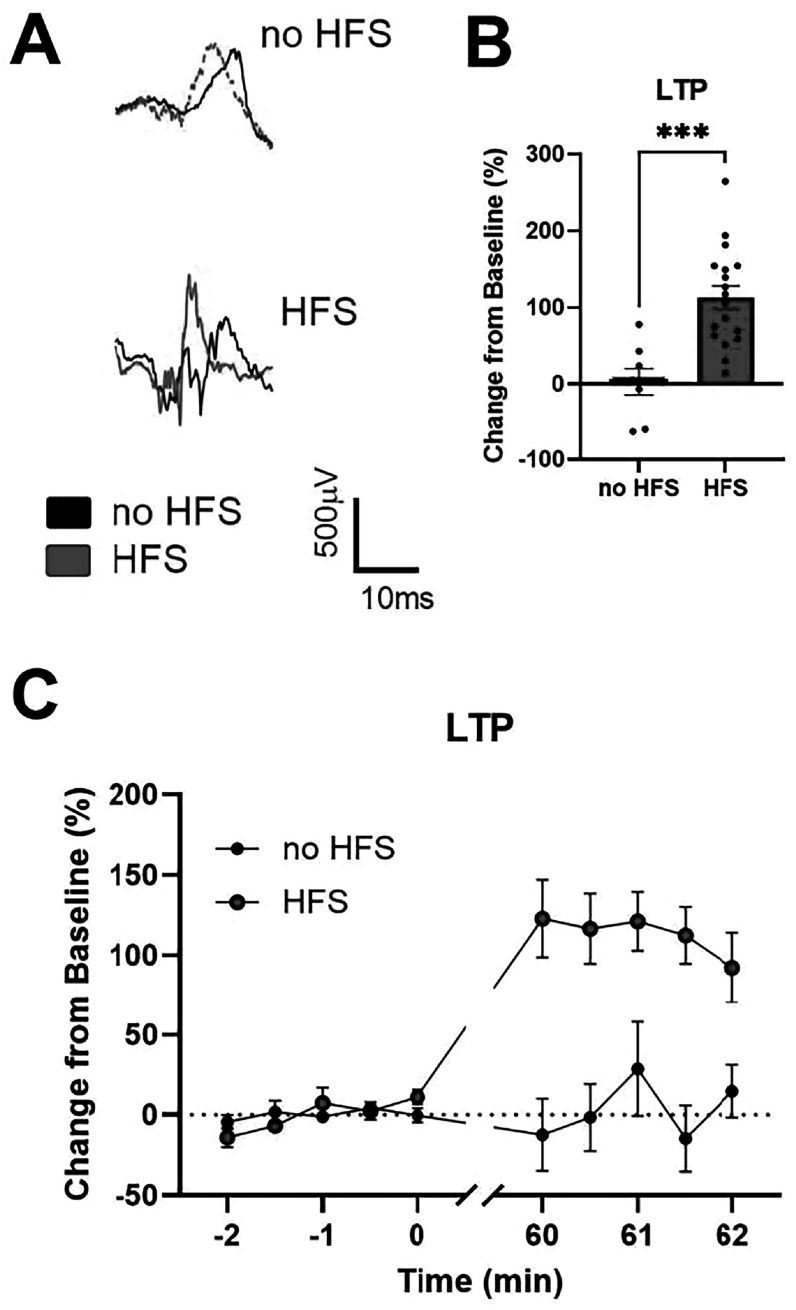
LTP is inducible without perfusion and without regular stimulation throughout the hour after induction, suggesting that sterile LTP recordings through SMEAs are possible. (A) Representative traces from electrodes in the CA1 of naïve OHSCs at baseline and at 1 h after receiving high frequency stimulation (HFS) or no HFS. (B) There is a significant increase in response magnitude due to HFS. Bar graph depicts the average of the last 5 stimulations of LTP normalized to the last 5 stimulations before induction. (C) Longitudinal graph of the 5 stimulations before induction with HFS and the last 5 stimulations after induction of LTP. Mean ± SEM; N = 8–18; ***p < 0.001 compared to no HFS using Student’s t-test.

We confirmed that LTP was inducible in sham samples through the SMEAs using a terminal protocol (figure 3). Previous experiments have stimulated an OHSC once a minute in the hour after induction of LTP. However, we sought to develop a new LTP-induction protocol which would not require perfusion, and would be the first step towards making sterile, serial recordings feasible. OHSCs that received no stimulation in the hour after induction of LTP with HFS were also able to potentiate (p < 0.001; figure 5). The evoked response was significantly larger in OHSCs that received HFS compared to OHSCs without HFS. Responses in OHSCs that did not receive HFS were similar to baseline (figure 5(B)). Average LTP recorded in cell culture medium in the naïve group was noticeably larger than LTP recorded in aCSF (figure 3).

### LTP was reduced on the second day of recording from previously potentiated samples

**Figure 6.**
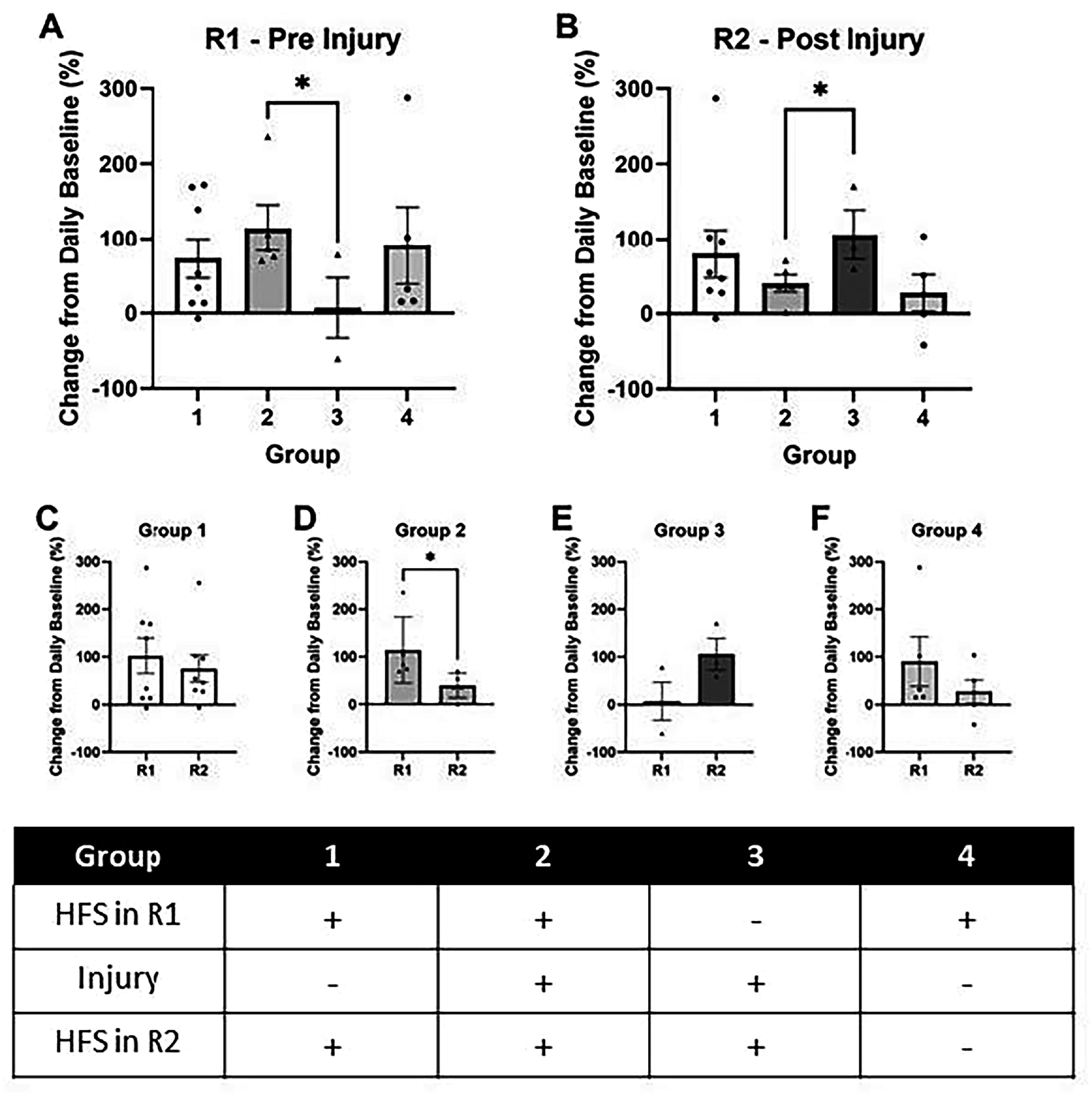
Serial LTP through SMEAs. (A) Results of recording 1 (R1) before injury where LTP was induced with high frequency stimulation (HFS) in groups 1, 2, and 4. (B) Results of recording 2 (R2) where LTP was induced with HFS in groups 1, 2, and 3. (C) Percent LTP in group 1, which was potentiated before and after sham-injury. Percent LTP was not significantly different between recordings 1 and 2. (D) Percent LTP in group 2, which was potentiated before and after injury. LTP was significantly decreased in R2 compared to R1. (E) Percent LTP in group 3, which was not potentiated before injury, but was potentiated after injury. (F) Percent LTP in group 4, which was potentiated before sham-injury and was not potentiated in R2. Table identifies treatments that each group received. Mean ± SEM; N = 3–8; *p < 0.05 using an unpaired one-tailed t-test in (A) and (B) and a paired one-tailed t-test in (D).

After developing an LTP protocol that did not require perfusion, this protocol was used to induce and measure LTP before and after injury. In the pre-injury recording, referenced as recording 1 (R1), LTP was induced in 3 of the 4 tested groups 24 h before stretch injury. There was no significant difference between the mean potentiation of these 3 groups. At 24 h after injury, in recording 2 (R2), LTP was induced in 3 of the 4 groups. There was a slight difference in potentiation between groups 2 and 3, which were both injured (p < 0.05; figure 6(B)).

The serial LTP protocol allowed us to compare R1 and R2 of the same samples within a group. For group 1, which was potentiated twice and sham-injured, there was no significant decrease in LTP during R2. However, in group 2, which was potentiated twice and injured, there was a significant decrease in LTP during R2 (p < 0.05; figure 6(D)). There were no significant changes between R1 and R2 for groups 3 and 4, which may be a result of low sample size and high variability.

## Discussion

TBI remains a major health concern worldwide [60]. To better understand post-traumatic deficits in function, we used a consistent mild injury defined by a biaxial tissue Lagrangian strain of approximately 15%. This data confirmed that we could impart strain values indicative of mild-to-moderate TBI on the OHSCs using SMEAs, which remained capable of stimulating and recording neuronal activity [22]. Lagrangian strains of up to 35% have been verified in OHSCs grown on silicone without embedded electrodes [51]. Although the strains achievable in this experiment are lower, the SMEAs can be used to record electrophysiological signals from mild-to-moderate injuries, giving each technology its own advantages.

In this study, we present differences in plasticity and neural network connectivity after mild stretch injury of OHSCs. When recording with glass MEAs, samples must be transferred to the electrodes from their culture substrate, which creates opportunities for the sample to be damaged. Stability of the recordings, even after mechanical deformation of the electrodes, highlights one benefit of the SMEA technology. Additionally, by using the SMEAs, LTP could be induced more than once in sham samples, providing a paradigm to test the interplay of synaptic strength and post-traumatic outcome.

We leveraged the abilities of the SMEAs to record spontaneous activity of the slices both before and at multiple time points after injury. Interestingly, at 2 h after injury, there were differences in the bursting behavior within the slice. Although the average bursting rate remained unchanged after injury, the average length of the burst increased. Additionally, because the firing rate remained unchanged, the percentage of events found within bursts also increased. Similar to our results, bursting behavior increases in the auditory cortex following TBI [61]. Bursting has been shown to be related to information processing [62], and recent hypotheses suggest that the information encoded in bursts differs from that of single spikes [63, 64].

Neurons have the ability to regulate their excitability to maintain a target level of activity. Typically, homeostatic plasticity regulates bursting and firing rate to prevent neurons from being over or under active [65]. Bursting often arises from high calcium influx, and a change in bursting behavior may indicate a shift in the excitatory/inhibitory balance in a cell [64, 66]. Post-injury disruption of calcium regulation may be responsible for the change in bursting activity observed after stretch injury [29, 66, 67].

Serial recordings of neuronal bursting after *in vitro* mechanical injury have been reported previously with a rigid MEA [68]. That model did not stretch the neuronal culture but used sheer stress from fluid flow to induce an injury. In that model, bursting rate decreased and the number of events within a burst decreased 10 min after injury. In contrast, we measured an increase in the number of events within a burst 2 h after injury, but this discrepancy could be due to the different time point studied. The immediate electrical responses after cell membrane perturbation could affect the activity after injury but may not last for 2 h; in future experiments, bursting immediately after stretch injury could be measured with our paradigm for comparison.

LTP and LTD are both forms of Hebbian plasticity, and deficits in one may impact the function of the other. Many of the same proteins needed for LTP are also involved in Ltd LTD is thought to be induced by lower frequency stimulation or slow and low calcium influx compared to LTP [69]. Recent work has suggested that LTD is also a form of learning. LTD is induced *in vivo* when novel objects are added to an environment, but not in urgent learning situations such as a foot shock test [70]. In our study, we measured deficits in both LTP and LTD 24 h after injury. There is no consensus about the effect of TBI on LTD [71]. Conflicting reports could be attributed to varying injury types and confounding systemic factors in animal models. Here, we employed an *in vitro* injury to quantify LTD following a purely mechanical stretch injury of isolated hippocampal tissue to limit confounding systemic factors.

In LTP, *α*-amino-3-hydroxy-5-methyl-4-isoxazolepropionic acid receptors (AMPARs) are recruited to the synapse and anchored with proteins including PSD95 and stargazin. In LTD, AMPAR are generally trafficked away from the synapse due to the dephosphoryation of stargazin and an increase in CaMKII activity [72, 73]. Peineau *et al* determined that LTP inhibited subsequent LTD at the same synapse, possibly due to the adverse effect of GSK3*β* [74]. Our previous work has reported, after injury, lower levels of PSD95 at baseline and an inability to increase stargazin and GluR1 AMPAR after LTP induction [75]. These same changes in protein expression could also affect LTD induction in the synapses. The LTP and LTD deficits at the same time point suggest that novel treatments should focus on proteins or pathways that are common between the two types of plasticity.

Although bursting behavior was increased 2 h after injury, it returned to baseline by 24 h when LTP and LTD deficits were measured in terminal experiments. Bursts have also been linked to synaptic plasticity, possibly because they can alter the membrane potential of a cell [66]. Bursting may itself modify synaptic plasticity. It is well established from groups, including ours, that stimulation with bursts at 100 Hz can induce LTP [26, 52, 76]. Although the bursting behavior did return to baseline by 24 h, the threshold for modifying synaptic strength could have been altered by earlier bursting [77, 78]. Based on previous studies by our group, LTP likely was not disrupted 2 h after injury, but that was not tested in this study or with stretch injury [75]. Cells can respond to increased release of neurotransmitters and the calcium influx believed to occur after injury, by decreasing their excitability [45, 67, 79]. Therefore, increased bursting activity after injury, and the subsequent cellular modifications required to suppress that activity, may be related to the deficits in Hebbian plasticity 24 h after injury. There may be more sensitive metrics of spontaneous activity that are disrupted 24 h after injury which could be identified in future studies.

Terminal LTP experiments required samples to be continuously stimulated during the progression of LTP. Serial LTP recordings were made possible by developing a new protocol with only intermittent stimulation. Electrophysiological recordings in cell culture medium are less common than in perfused aCSF, but LTP induction has been reported in culture medium previously [80, 81]. Interestingly, we found that potentiation was larger in samples submerged in medium compared with samples in aCSF, a finding corroborated in the literature [52]. The frequency of stimulation may affect LTP progression, with increased stimulation frequency leading to reduced LTP induction [82, 83]. It is not clear from these experiments if differences in potentiation were due to the altered stimulation protocol or the choice of aCSF versus cell culture medium during recording.

*In vivo*, neurons continuously alter their synaptic strength to modulate their activity in response to inputs. In our model, it was possible to re-induce LTP 48 h after initially inducing LTP. The ability to re-induce LTP in the same slice indicates that the increased synaptic strength of the first LTP induction did not endure until the second electrophysiological recording. Our data is contrary to the work published by Shimono *et al*, in which potentiation lasted several days after 3 rounds of 100 pulses at 100 Hz, but with 10 min inter-train intervals rather than 10 seconds as in our paradigm [84]. Additionally, in an experiment with human iPSCs, LTP was induced chemically to last at least 48 h [85]. However, different induction protocols can induce different forms of LTP [86]. Repeated induction of LTP and LTD have been attempted before in OHSCs [87, 88]. Ogura’s group used forskolin to induce LTP [89]. A single administration of forskolin induced LTP that lasted less than 24 h. However, 3 administrations of forskolin within 24 h produced changes in synaptic response that lasted for weeks [88–90]. It seems that, in our model, an induction protocol of 3 bursts of 100 pulses at 100 Hz separated by 10 seconds was not sufficient to induce LTP enduring until the second recording 48 h later.

After injury, LTP on the second day of recording was decreased compared to the first induction, which is in agreement with the terminal LTP data in this study. Although this data is preliminary, the OHSCs that did not receive HFS before injury potentiated more than the group that did receive HFS before injury. This is an interesting divergence from the results gathered with terminal LTP recordings. It is not clear from these experiments why mild injury could cause deficits in LTP in a terminal experiment but not a serial experiment. It is possible that the altered ion or glucose concentrations in the culture medium compared to the aCSF were responsible for different firing dynamics. The glucose concentration in our cell culture medium is at least 125% of the glucose concentration in aCSF, and glucose administration increases LTP [91, 92]. Additionally, while LTP is reduced following injury with HFS in aCSF, LTP may be inducible with more robust LTP stimulation, as is seen in aged rats [93]. It is possible that the increased glucose in the recording solution made for a more robust LTP induction. In the future, a different and shorter stimulation protocol could be used to identify an injury effect in samples that did not receive HFS before injury. However, this paradigm allowed us to observe the preliminary finding that previous HFS stimulation decreased LTP.

Although complex, the data suggests that LTP induction before injury hampered recovery. One possibility is that previously strengthened synapses, with more AMPAR inserted into the synapse, were more susceptible to damage from post-traumatic glutamate release, which can occur even in mTBI [94]. In a similar vein, antagonism of mGluR5 acts to decrease the clearance of glutamate from the synaptic cleft, which leads to increased extracellular glutamate. mGluR5 agonists facilitate LTP, and antagonists can inhibit LTP [95]. The benefits of mGluR5 modulation are controversial as a TBI treatment, with studies reporting benefits of both agonists and antagonists. Overall, the majority of studies antagonizing mGluR5 receptors before injury elicited neuroprotection [44, 96, 97]. However, other studies have found benefits of mGluR5 agonism when administered after injury [97–99]. Pre-treatment with inhibitors of mGluR5 may be beneficial because the network becomes more resilient to injury following LTP inhibition. However, more work is needed to identify if this difference is due to a decreased susceptibility to glutamate overexposure or another mechanism.

More broadly, there are still many unknowns regarding why some patients recover from a mild TBI and why some have longer lasting symptoms. Results of this study may be a first step to predicting patient prognosis. Our data suggests that previously potentiated neurons were more susceptible to injury than non-potentiated neurons. The mechanisms of LTP in healthy neurons are well established, and our work could inform potential biomarkers that could be used to predict recovery. Additionally, medical imaging, such as fMRI, after trauma could be used to approximate network connectivity, potentially indicate prognosis, and influence treatment decisions. Information from these LTP and LTD studies could partially inform imaging in the future.

Our model is not without limitations. These experiments employed an *in vitro* model of the hippocampus in isolation from other regions of the brain and only studied 2 acute time points after injury. Additionally, the sparseness of the SMEAs made quantifying network connectivity challenging. This work presented preliminary evidence that network state before injury may influence network recovery after injury, but more work is needed to investigate further. Future work could expand on the serial LTP data to better understand the time course of plasticity deficits after mild injury. Furthermore, if the density of the electrodes on SMEAs in increased, future work could more precisely measure and alter the network state before injury.

## Data Availability

The data cannot be made publicly available upon publication because the cost of preparing, depositing and hosting the data would be prohibitive within the terms of this research project. The data that support the findings of this study are available upon reasonable request from the authors.

## Significance statement

Deficits in neuronal plasticity (LTP and LTD) and alterations in spontaneous activity following *in vitro* mild traumatic brain injury are reported. These studies were enabled by a novel stretchable microelectrode array that is compatible with long-term tissue culture, large biaxial deformation, serial recordings, and robust electrical stimulation. We provide preliminary evidence that altering the neuronal network organization before injury by inducing LTP was detrimental to neuronal network function post-injury. Future studies will continue to explore how modulation of the neuronal network before injury affects post-traumatic recovery.
